# Neurological Manifestations of Acute SARS-CoV-2 Infection in Pediatric Patients: A 3-Year Study on Differences between Pandemic Waves

**DOI:** 10.3390/v16060967

**Published:** 2024-06-17

**Authors:** Iolanda Cristina Vivisenco, Andreea Lescaie, Ana Dragomirescu, Ioana Cătălina Ioniță, Irina Florescu, Bogdan Ciocea, Andreea Rodica Grama, Maria-Dorina Crăciun, Carmen-Daniela Chivu, Coriolan Emil Ulmeanu, Viorela Gabriela Nițescu

**Affiliations:** 1 Discipline of Pediatrics, Faculty of Dentistry, Carol Davila University of Medicine and Pharmacy, 17-23 Plevnei Road, 010221 Bucharest, Romania; cristina.vivisenco@umfcd.ro (I.C.V.); rodica-andreea.grama@umfcd.ro (A.R.G.); coriolan.ulmeanu@umfcd.ro (C.E.U.); viorela.nitescu@umfcd.ro (V.G.N.); 2 Department of Pediatrics, Grigore Alexandrescu Clinical Emergency Hospital for Children, 30-32 Iancu de Hunedoara Road, 011743 Bucharest, Romania; ana.dragomirescu@rez.umfcd.ro (A.D.); ioana-catalina.ionita@rez.umfcd.ro (I.C.I.); 3 Department of Neurology, Grigore Alexandrescu Clinical Emergency Hospital for Children, 30-32 Iancu de Hunedoara Road, 011743 Bucharest, Romania; flores_irina@yahoo.com (I.F.); bogdan.ciocea@gmail.com (B.C.); 4 Department of Epidemiology, Faculty of Medicine, Carol Davila University of Medicine and Pharmacy, 17-23 Plevnei Road, 010221 Bucharest, Romania; maria.craciun@umfcd.ro (M.-D.C.); carmen-daniela.chivu@drd.umfcd.ro (C.-D.C.); 5 Department of Infection Prevention and Control, Grigore Alexandrescu Clinical Emergency Hospital for Children, 30-32 Iancu de Hunedoara Road, 011743 Bucharest, Romania

**Keywords:** COVID-19, SARS-CoV-2, pandemic waves, children, neurologic manifestations, Omicron

## Abstract

This study analyzed the neurological manifestation profiles of severe acute respiratory syndrome coronavirus 2 (SARS-CoV-2) infection across pandemic waves in pediatric patients. The study collected data on patients aged between 0 and 18 years, diagnosed with acute SARS-CoV-2 infection, admitted to a pediatric tertiary hospital between 1 March 2020 and 28 February 2023. This study included 1677 patients. Neurological manifestations were noted in 10% (n = 168) of patients with a median age of 3.2 years (interquartile range: 1–11.92). Neurological manifestations were significantly associated with the pandemic waves (*p* = 0.006) and age groups (*p* < 0.001). Seizures were noted in 4.2% of cases and reached an increasing frequency over time (*p* = 0.001), but were not associated with age groups. Febrile seizures accounted for the majority of seizures. Headache was reported in 2.6% of cases and had similar frequencies across the pandemic waves and age groups. Muscular involvement was noted in 2% of cases, reached a decreasing frequency over time (*p* < 0.001), and showed different frequencies among the age groups. Neurological manifestations of acute SARS-CoV-2 infection exhibit distinct patterns, depending on the pandemic wave and patient age group. The Wuhan and Omicron waves involved the nervous system more often than the other waves.

## 1. Introduction

The severe acute respiratory syndrome coronavirus 2 (SARS-CoV-2) pandemic has received widespread attention from healthcare professionals and researchers worldwide [1,2]. The likelihood of pediatric patients contracting SARS-CoV-2 was lower in comparison to adults, and they generally exhibited either asymptomatic or milder symptoms [3,4,5,6,7]. Nevertheless, there were instances in which children experienced severe and atypical illnesses [4].

The field of neurology has generated significant interest in relation to SARS-CoV-2 infection, with over 5000 original articles and reviews published on this topic [8,9,10,11]. The occurrence of neurological manifestations in coronavirus disease 2019 (COVID-19) was not surprising, as Middle East respiratory syndrome and severe acute respiratory syndrome coronavirus 1 have been associated with a variety of neurological signs and symptoms [12,13,14]. However, SARS-CoV-2 was associated with neurological manifestations that were not commonly reported with other coronaviruses [15].

It was initially believed that neurological manifestations were rare in pediatric patients [16,17,18]. As research has pointed out the range of neurological symptoms, it has become evident that these symptoms occur frequently in children as well, although their types differ substantially from those identified in adults [19,20,21,22,23,24]. It is worth noting that children generally displayed a higher frequency of seizures, whereas adults were more prone to experiencing strokes [19,20,21,22,23,24].

The neurological presentations of SARS-CoV-2 variants may differ [25]. To the best of the authors knowledge, there is a lack of studies investigating all variants of concern for variant-specific neurological manifestations, despite the necessity of such research [26]. Therefore, this study aimed to provide a thorough characterization of the neurological manifestations associated with all SARS-CoV-2 variants of concern reported in the pediatric population.

## 2. Materials and Methods

### 2.1. Study Design

This study analyzed patients under 18 years of age diagnosed with acute SARS-CoV-2 infection who were admitted to a pediatric hospital between 1 March 2020, and 28 February 2023. This study conformed to the ethical principles of the Declaration of Helsinki for medical research. The guardians of all the patients provided informed consent for participation in the study. Approval was obtained from the Ethical Committee of the hospital prior to the commencement of the study (8336/20 March 2023).

The electronic medical records of the hospital informatic system were screened using the keywords “COVID-19”, “SARS-CoV-2”, and U07.1 (COVID-19, virus identified) code of the International Statistical Classification of Diseases and Related Health Problems, Tenth Revision, Australian Modification (ICD-AM-10). According to the national COVID-19 case definition adapted from the World Health Organization (WHO), each patient enrolled in the study was mandated to have a confirmed SARS-CoV-2 infection through either a rapid antigen test or polymerase chain reaction (PCR) test [27,28]. Patients diagnosed with COVID-19 within 90 days of an initial positive test were counted as one occurrence to reduce prolonged positive test results.

The medical records of each patient (n = 1714) were subjected to rigorous examination by a panel of two pediatrician investigators to determine whether neurological symptoms were present at the time of admission or during hospitalization. Subsequently, a team of two pediatric neurologists reviewed the association between neurological manifestations and SARS-CoV-2 infection. Consequently, two groups were defined: the first group included patients with at least one new-onset neurological symptom or exacerbation of a pre-existing neurological condition, while the second group included patients without neurological symptoms. 

It was impossible to retrospectively establish the exact ages of 37 patients because they were foreign citizens with refugee status. Consequently, these cases were excluded, and it should be noted that none of these patients exhibited any neurological symptoms.

The primary objective of this study was to assess the variation in the frequency of neurological manifestations across the waves of the SARS-CoV-2 pandemic in Romania. The secondary objectives were to identify the frequency and nature of the neurological manifestations in children infected with SARS-CoV-2 and to evaluate their variations across different age groups.

### 2.2. Data Collection

Data from electronic medical records were recorded on a spreadsheet using a pre-established coding system according to the recommendations of McNett et al. [29]. The extracted information included demographic characteristics, date of admission, pre-existing neurological diseases, clinical manifestations exhibited at the time of admission and throughout the hospitalization period, and paraclinical features (SARS-CoV-2 rapid antigen or PCR test, pressure and composition of cerebrospinal fluid, cerebral imaging studies, and electroencephalogram).

Data regarding the national reports of SARS-CoV-2 genomic sequences were obtained from the Global Data Sharing Initiative [30,31] and the National Institute of Public Health [32]. The national reports of individuals with COVID-19 by age group were obtained from the WHO COVID-19 detailed surveillance data dashboard [33].

### 2.3. Definitions

The pandemic wave term describes a period within the occurrence of a specific SARS-CoV-2 variant that was detected in over 50% of the genomic sequences analyzed in Romania (Table 1). The first wave of the virus did not receive an official designation from the WHO and has been referred to in previous research as Wuhan, Wild-type, or the original strain. In this study, the Wuhan label was used.

Patients were categorized according to age based on the WHO global reports, which included age groups ranging from 0 to 4, 5 to 9, 10 to 14, and 15 to 19 years [33]. However, since the study center only admitted individuals under the age of 18, no patients aged 19 years were included in the study. As a result, the respective age group was named 15 to 18 years.

The new-onset neurological manifestations included in this study were seizures, headache, muscular involvement, vasovagal syncope, apnea, anosmia, dysgeusia, encephalopathy, idiopathic endocranial hypertension, ischemic stroke, ataxia, peripheral neuropathy, and sleep myoclonus. The case definitions for these manifestations were in accordance with the scientific literature and are provided in Table A1 of Appendix A [34,35,36,37,38,39,40,41,42,43,44,45,46].

Exacerbation of a pre-existing neurological condition was considered to be the manifestation of symptoms associated with the underlying disease in an individual who received treatment for that disease.

### 2.4. Statistical Analysis

All statistical analyses were performed using the XL-STAT version 2023.5 (Addinsoft, Paris, France) and VassarStats version SCR-010263 (Vassar College, New York, NY, USA) software. The relationships between neurological features and pandemic waves or age groups were assessed using the chi-square test (Yates correction [47]). When the criteria for reporting the chi-square test results were not met, Fisher’s exact two-tailed test (Freeman–Halton extension [48]) was performed. The trend of neurological manifestation frequencies among age groups or pandemic waves was assessed using the Cochran–Armitage test [49,50]. Standardized residual analyses were performed, and the results were reported as z-scores (z) [51]. The significance level was set at a *p*-value of less than 0.05, with an Alpha risk of 5%.

The study used Pearson correlation analysis to assess whether the number of patients hospitalized monthly at the study center was representative of the Romanian population aged between 0 and 19 years infected with SARS-CoV-2.

## 3. Results

This study comprised 1677 cases of acute SARS-CoV-2 infection, with a 10% (n = 168) frequency of neurological manifestations. The study sample accurately represented the frequency of COVID-19 in Romania for patients under 19 years of age, showing a strong correlation between the monthly number of hospitalized patients at the study center and the cumulative number of patients in Romania (Table 2).

### 3.1. All Neurological Manifestations

The age range of patients with neurological manifestations during acute SARS-CoV-2 infection was broad, from 9 days to 17.58 years of age, with a median age of 3.2 years (interquartile range: 1–11.92).

Neurological symptoms appeared within the first 24 h of respiratory or digestive issues in 58% of cases (98/168), between 24 and 48 h in 2% (n = 3), between 48 and 72 h in 8% (n = 14), and after more than 72 h in 9% (n = 15) of cases. Seven patients (4%) did not experience respiratory or digestive issues at the onset of neurological symptoms, and the temporal relationship between these symptoms was not documented in the remaining 18% (n = 31).

**Figure 1:**
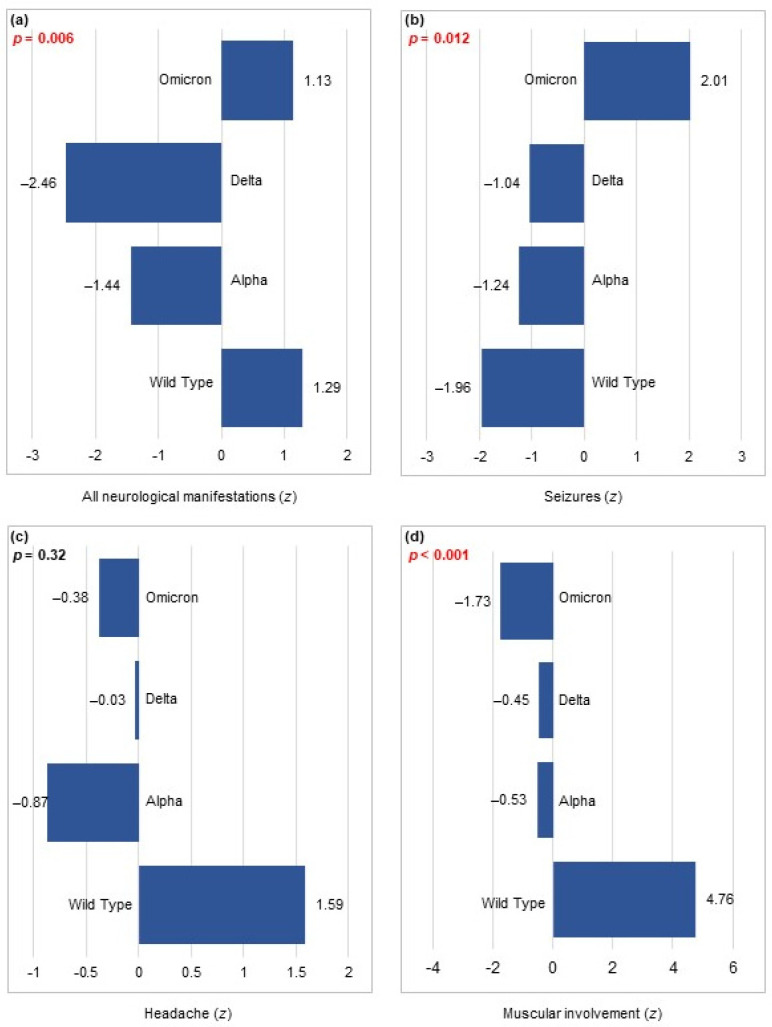
The association between pandemic waves and (**a**) all neurological manifestations (n = 168), (**b**) seizures (n = 71), (**c**) headaches (n = 43), and (**d**) muscular involvement (n = 34) in the study population (n = 1677).

The association between neurological manifestations and SARS-CoV-2 waves was statistically significant (*p* = 0.006); however, no time trend was observed (Table 3). As shown in Figure 1a, the frequency of these manifestations was higher during the Wuhan and Omicron waves.

**Figure 2:**
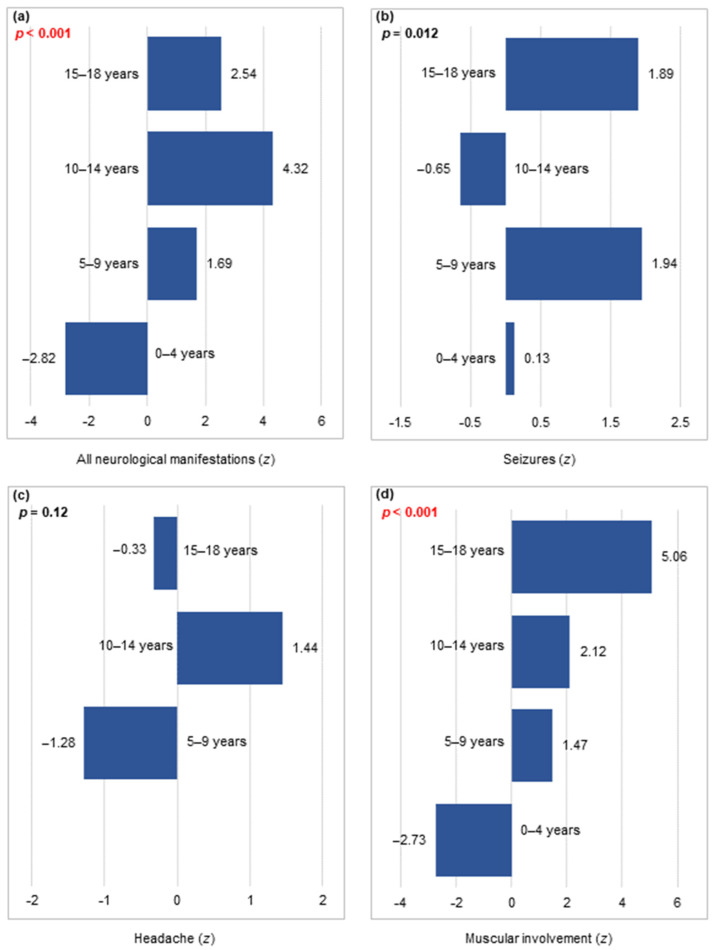
The association between age groups and (**a**) all neurological manifestations (n = 168), (**b**) seizures (n = 71), (**c**) headache (n = 43), and (**d**) muscular involvement (n = 34) in the study population (n = 1677).

Neurological symptoms showed a relationship with age groups (*p* < 0.001; Figure 2a), with the frequency increasing with age (*p* < 0.001; Table 4). Moreover, neurological manifestations were significantly associated with SARS-CoV-2 waves in patients aged between 0 and 4 years (χ^2^(3) = 8.05, *p* = 0.045; Table A2 of the Appendix A).

### 3.2. Seizures

Seizures were documented in 4.2% (n = 71) of cases, with 54 instances being febrile seizures. Five patients experienced new-onset seizures without fever, of whom only one was subsequently diagnosed with epilepsy, whereas the others had a normal electroencephalogram. Seizures occurred as an exacerbation of pharmacologically controlled epilepsy in 12 (0.7%) cases, three of which developed status epilepticus.

Statistical analysis revealed a notable disparity in the occurrence of seizures across the various waves of the COVID-19 pandemic (*p* = 0.012), with a higher frequency observed as new waves emerged (*p* = 0.001; Table 3). Figure 1b shows the trend of seizure frequency, which was lowest in the Wuhan wave (z = −1.96) and highest in the Omicron wave (z = +2.01).

Seizures were not associated with age group, and no trend was observed (Table 4). Analysis of seizures across SARS-CoV-2 waves within each age group showed a significant time trend for patients aged between 0 and 4 years (χ^2^(1) = 6.32, *p* = 0.012; Table A2 of Appendix A).

### 3.3. Febrile Seizures

Of the total number of patients diagnosed with seizures, 54 experienced febrile seizures during the acute SARS-CoV-2 infection. Of these, 37 were diagnosed with simple febrile seizures, 13 with complex febrile seizures, and four with febrile status epilepticus.

The frequency of febrile seizures reached a minimum in the Wuhan wave and a maximum in the Omicron wave. Febrile seizures were significantly associated with SARS-CoV-2 waves (*p* = 0.010), with a clear trend over time (*p* = 0.002; Table 3).

Analysis of seizures across SARS-CoV-2 waves within each age group showed a significant time trend for patients aged between 0 and 4 years (χ^2^(1) = 6.24, *p* = 0.013; Table A2 of Appendix A).

### 3.4. Headache

Headache was reported in 43 patients (2.6%), with a similar frequency between the SARS-CoV-2 waves (Table 3). However, it is worth noting that the highest frequency was registered during the Wuhan wave (Figure 1c).

The analysis of headaches across age groups showed that the highest frequency was reported for patients aged between 10 and 14 years (Figure 2c), although a statistically significant difference was not observed (Table 4).

### 3.5. Muscular Involvement

Muscular involvement was reported in 2% (n = 34) of cases, including myalgia in 23 cases, hypotonia in six, and myositis in five.

A significant correlation was observed between muscular involvement and the various waves of the pandemic (*p* < 0.001), which also demonstrated a time trend (*p* < 0.001; Table 3). Notably, the highest frequency of muscular involvement was recorded during the Wuhan wave, whereas the lowest frequency was observed during the Omicron wave (Figure 1d).

The frequency of muscular involvement was significantly different among the age groups (Figure 2d) and increased with age (Table 4).

### 3.6. Other Neurological Manifestations

Vasovagal syncope occurred in 1.1% (n = 18) of the patients, out of which 10 were triggered by fever. All cases occurred in Wuhan (n = 5) or in Omicron waves (n = 13). An age-repartition analysis revealed that nine patients were in the 0 to 4 age group, two were in the 5 to 9 age group, four were in the 10 to 14 age group, and three were in the 15 to 18 age group.

Apnea was documented in 0.7% (n = 12) of cases, being a solitary event in nine patients and a recurrent occurrence in three. The age of the patients affected by apnea was below 4 months, except for a 1-year-old and a 4-month-old girl with Aicardi syndrome.

Anosmia and dysgeusia were reported in seven patients (0.4%) diagnosed during the Wuhan and Delta waves. The ages of the patients ranged from 8 to 17 years old.

Encephalopathy was documented in 0.4% (n = 7) of the cases and was reported to affect patients aged between 1 month and 5 years.

Idiopathic intracranial hypertension was reported during the Omicron wave in five infants (0.3%) aged between 2 and 9 months.

An ischemic stroke was diagnosed in a 2-month-old male infant born to a mother with thrombophilia and hospitalized for an acute SARS-CoV-2 infection.

Certain neurological disorders were documented in only one patient: ataxia during the Omicron wave in a 13-year-old boy, peripheral neuropathy during the Omicron wave in an 11-year-old girl, and sleep myoclonus during the Delta wave in an 11-year-old boy.

Exacerbations of pre-existing conditions occurred in children with epilepsy (n = 13), myasthenia gravis (n = 1), and multiple sclerosis (n = 1).

## 4. Discussion

The findings of this study showed that 10% of pediatric patients with acute SARS-CoV-2 infection experienced neurological manifestations. Although the neurological manifestations were consistent across all pandemic waves, the frequency of these manifestations varied based on the wave of the pandemic and the age of the patient. The neurological manifestations described in this study have been documented in scientific literature, including rare cases [52,53,54,55,56,57,58,59,60,61,62,63].

The neurological consequences of COVID-19 according to age have been explored in several studies with varying results [64,65,66]. The literature review revealed inconsistencies in the classification of age and the absence of age groups used in the official WHO reports. However, this study adopted the same age groups as those presented in the WHO’s global reports on COVID-19 to facilitate data comparisons that may arise in future research in this area [33].

The frequency of neurological manifestations in pediatric patients with acute SARS-CoV-2 infection was lower in this study (10%) than in most previous studies (13.5–40%) [19,67,68,69], but was in line with the findings of Antoon et al. [64] (7%). The frequency of neurological manifestations in pediatric patients may vary due to differences in methodologies, such as variations in upper age limits, analyzed period, and inclusion of multisystem inflammatory syndrome.

Proust et al. [70] identified distinct patterns of central nervous system (CNS) cell invasion in Wuhan and Omicron variants, which may explain the different neurological manifestations associated with each variant. To the best of our knowledge, this is the only real-world study currently available to validate the findings of Proust et al. [70] regarding the increased neurological involvement of the Wuhan and Omicron variants compared with the Alpha and Delta variants.

Studies that analyzed the neurological manifestations during the Omicron wave and compared them with previous periods or other SARS-CoV-2 variants found evidence to support the increase in neurological symptoms [66,71,72,73]. In contrast, Antoon et al. [64] noted more neurological manifestations during the Wuhan wave than during the Omicron wave. This difference may be due to the shorter Omicron spread considered by Antoon et al. [64].

Seizures, including de novo status epilepticus, have been frequently reported in pediatric patients with COVID-19 [74,75]. Moreover, some studies have indicated that seizures are the most common neurological manifestations [66]. The reported frequencies varied across studies, ranging from 0.06% to 61.9% [12,66]. The present study showed a striking increase in seizure frequency during the Omicron wave compared to prior waves. This result was unexpected, as Omicron was believed to be less severe than prior variants [10]. According to Cho et al. [76], seizure frequency decreases with age. However, our study revealed that the highest frequency of seizures was observed in the 5–9 age group (7.8%).

This study, as well as others [64,73], discovered that febrile seizures were the most common neurological manifestation among pediatric patients diagnosed with COVID-19. It is worth noting that Kim et al. [77] highlighted the potential of COVID-19 to cause more severe febrile-induced seizures.

Previous studies have reported headache frequencies ranging from 10% to 20% [12,76], while this study found a frequency of 2.6%. This disparity may be attributable to the subjective nature of headaches and the inability of young children to communicate this symptom.

It is important to note that this study revealed a lower frequency of muscular involvement in patients with COVID-19 than previously reported [12,76]. This discrepancy may be attributed to variations in clinical examination techniques.

This study had several strengths. First, although this study is not the first to evaluate the neurological manifestations of SARS-CoV-2 among various waves or age groups, it has the longest study duration among similar studies. Second, this study involved a comprehensive comparison of all the prevailing SARS-CoV-2 variants in Romania. Third, although our study is limited by single center data, it has the merit of including a cohort that reflects the dissemination of COVID-19 in Romania.

This study has certain limitations. The inclusion of only inpatients may lead to underreporting of neurological symptoms in children with mild or asymptomatic COVID-19. Furthermore, the study may underestimate the self-reported symptoms, especially in younger children who cannot communicate subjective sensations. The reported frequencies may also have been influenced by the retrospective and unicentric nature of this study. Additionally, the specific SARS-CoV-2 variant for each patient could not be determined, as they were categorized based on the prevalent variant in Romania during the corresponding period. It should also be noted that the neurological symptoms detailed in this study are not specific to acute infection with SARS-CoV-2.

This study highlights the necessity of conducting multicenter studies with similar methodologies to gain a comprehensive understanding of the age- and variant-specific neurological manifestations of acute SARS-CoV-2 infection. Such studies can contribute to understanding the potential risks associated with long COVID-19 syndrome.

## 5. Conclusions

Neurological manifestations of acute SARS-CoV-2 infection exhibited distinct patterns depending on the pandemic wave and patient age group. The first and last waves (Wuhan and Omicron) displayed a greater tendency towards neurological manifestations when compared to the other waves. However, the profiles of the neurological manifestations varied between these two waves. While seizures were more prevalent in the Omicron wave, headaches, and muscular involvement were more common in the Wuhan wave. This observation aligns with prior research that has highlighted the distinct mechanisms of neurological involvement associated with the Wuhan and Omicron variants.

## Figures and Tables

**Table 1:** SARS-CoV-2 pandemic waves in Romania.

Pandemic Wave	Period
Wuhan	1 March 2020—31 December 2020
Alpha	1 January 2021—30 June 2021
Delta	1 July 2021—31 December 2021
Omicron	1 January 2022—28 February 2023

**Table 2:** Pearson coefficient of association between the monthly hospitalized patient count at the study center and the national count among age groups.

Age Group	COVID-19 Patients at Study Center		COVID-19 Patients at National Level		Pearson *r* (95% CI)	*p* Value
	Total (n)	Monthly Mean (SD)	Total (n)	Monthly Mean (SD)		
0–4 years	1276	32.8 (46.3)	88151	3896.4 (2448.6)	0.88 (0.72–1.05)	<0.001
5–9 years	129	3.1 (4.6)	76237	2117.7 (4434.62)	0.76 (0.54–0.99)	<0.001
10–14 years	158	3.5 (4.7)	121987	3388.5 (7378.4)	0.80 (0.59–1.01)	<0.001
15–19 years	114	2.6 (2.9)	117102	3252.8 (5901.2)	0.54 (0.25–0.84)	0.001
All patients	1677	41.9 (56.5)	403477	11207.7 (20929)	0.79 (0.58–1.01)	<0.001

**Table 3:** Neurological manifestation frequency and their association with the pandemic waves.

	Wuhan Wave (n = 208)	Alpha Wave (n = 143)	Delta Wave (n = 355)	Omicron Wave (n = 971)	χ^2^(3) (*p* Value)	χ^2^(1) (*p* Value)
All neurological manifestations % (n)	12.5 (26)	3.5 (5)	7.6 (27)	11.3 (110)	12.31 (0.006)	0.83 (0.36)
Seizures % (n)	1.4 (3)	2.1 (3)	3.1 (11)	5.6 (54)	10.96 (0.012)	10.15 (0.001)
Febrile seizures % (n)	1 (2)	1.4 (2)	2 (7)	4.4 (43)	11.25 (0.010)	9.80 (0.002)
Headache % (n)	4.3 (9)	1.4 (2)	2.5 (9)	2.4 (23)	3.51 (0.32)	1.24 (0.27)
Muscular involvement % (n)	6.7 (14)	1.4 (2)	1.7 (6)	1.2 (12)	26.71 (<0.001) *	18.95 (<0.001) *

Note: * These values should be interpreted with caution as they do not meet the reporting criteria for the chi-square test.

**Table 4:** Neurological manifestation frequency and their association with the age groups.

	0–4 Years (n = 1276)	5–9 Years (n = 129)	10–14 Years (n = 158)	15–18 Years (n = 114)	χ^2^(3) (*p* Value)	χ^2^(1) (*p* Value)
All neurological manifestations % (n)	7.5 (96)	14.7 (19)	20.9 (33)	17.5 (20)	39.85 (<0.001)	34.25 (<0.001)
Seizures % (n)	4.3 (55)	7.8 (10)	3.2 (5)	0.9 (1)	7.57 (0.056)	1.85 (0.17)
Febrile seizures % (n)	3.7 (47)	4.7 (6)	0 (0)	0.9 (1)	8.99 (0.029) *	6.04 (0.014) *
Headache % (n)	NA	6.2 (8)	13.3 (21)	8.8 (10)	4.23 (0.12) ^	4.23 (0.12)
Muscular involvement % (n)	0.9 (12)	3.9 (5)	4.4 (7)	8.8 (10)	40.51 (<0.001) *	38.78 (<0.001) *

Note: * These values should be interpreted with caution as they do not meet the reporting criteria for the chi-square test. ^ This value represents the chi-square test results with two degrees of freedom, not three as in the other cells.

## Data Availability

The data that support the findings of this study are available from the corresponding author upon reasonable request due to privacy concerns.

## Appendix A

**Table A1:** Definition used for diagnosis of new-onset neurological manifestations during acute SARS-CoV-2 infection.

Neurological Manifestation	Definition
Seizures	A paroxysmal manifestation involving sudden loss of consciousness and uncontrollable movements, or generalized hypotonia. A pediatric neurologist determined the diagnosis through medical history and clinical presentation.
Simple febrile seizures	A single episode of generalized seizures occurring within a 24-h period and lasting less than 15 min, in the presence of focal signs accompanied by a fever (temperature above 38 °C) in children under 5 years of age without any underlying neurological diseases [34,35].
Complex febrile seizures	Seizures that occur in children with an underlying neurological disease or prolonged (over 15 min), focal, or multiple seizures in a 24-h period in children without any underlying neurological diseases. These seizures are accompanied by a fever [35].
Established status epilepticus	Continuous seizures or recurrent seizures without consciousness recovery over a period of 30 min or more in children who do not have fever [36,37,38].
Febrile status epilepticus	Continuous seizures or recurrent seizures without consciousness recovery over a period of 30 min or more, accompanied by fever [35].
Headache	A clinical diagnosis was made after the exclusion of any additional underlying disorders other than SARS-CoV-2 infection in patients who reported headaches during medical examination.
Muscular involvement	Myalgia or hypotonia reported by the patient or detected by the pediatrician during the medical examination.
Vasovagal syncope	A temporary, non-traumatic loss of consciousness is triggered by certain factors [39].
Apnea	Interruption of breathing for a brief period, accompanied by bradycardia, cyanosis, or pallor [40].
Anosmia	Partial or complete loss of smell was reported by the patient or patient guardian during the medical examination.
Dysgeusia	Taste disturbances reported by the patient or patient guardian during a medical examination.
Encephalopathy	A diffuse brain condition characterized by altered mental status (lethargy, reduced consciousness, or changes in personality) that persist for over 24 h. The diagnosis was established after the exclusion of any underlaying disorder other than SARS-CoV-2 infection [41,42].
Idiopathic endocranial hypertension (suggested)	Signs and symptoms of increased intracranial pressure, with a normal composition of cerebrospinal fluid, and normal brain imaging [43].
Ischemic stroke	Sudden onset of neurological symptoms such as hemiplegia or any focal neurological signs, followed by the identification of ischemic stroke through cerebral imaging.
Ataxia	The disruption of posture and movement coordination. The diagnosis was made by a pediatric neurologist through a medical history and clinical presentation [44].
Peripheral neuropathy	Self-limited symptoms of peripheral motor or sensory nerve fibers were reported during a medical examination [45].
Sleep Myoclonus	Involuntary and benign brief movements that occur during sleep. The diagnosis was made by a pediatric neurologist based on clinical presentation after ruling out other medical conditions [46].

**Table A2:** Frequency of neurological manifestations and their association with pandemic waves for each age group.

	Wuhan Wave %, n	Alpha Wave %, n	Delta Wave %, n	Omicron Wave %, n	χ^2^(3) (*p* Value)	χ^2^(1) (*p* Value)
All neurological manifestations						
0–4 years	6.6 (9)	2.9 (3)	5.2 (13)	9.1 (71)	8.05 (0.045)	4.12 (0.043)
5–9 years	14.3 (2)	0 (0)	7.1 (2)	19 (15)	3.81 (0.28) *	1.35 (0.25) *
10–14 years	22.2 (6)	5.3 (1)	22.5 (9)	23.6 (17)	3.22 (0.36) *	0.54 (0.46) *
15–18 years	29 (9)	8.3 (1)	8.6 (3)	19.4 (7)	5.57 (0.13)	1.23 (0.27)
Seizures						
0–4 years	1.5 (2)	2.9 (3)	2.8 (7)	5.5 (43)	7.23 (0.06)	6.32 (0.012)
5–9 years	0 (0)	0 (0)	7.1 (2)	10.1 (8)	2.49 (0.48) *	2.37 (0.12) *
10–14 years	3.7 (1)	0 (0)	2.5 (1)	4.2 (3)	0.94 (0.82) *	0.17 (0.68) *
15–18 years	0 (0)	0 (0)	2.9 (1)	0 (0)	2.28 (0.52) *	0.08 (0.78) *
Febrile seizures						
0–4 years	1.5 (2)	1.9 (2)	2 (5)	4.9 (38)	7.38 (0.050)	6.24 (0.013)
5–18 years	0 (0)	0 (0)	1.9 (2)	2.7 (5)	2.93 (0.40) *	2.76 (0.10) *
Headache						
5–9 years	7.1 (1)	0 (0)	3.6 (1)	7.6 (6)	1.15 (0.77) *	0.24 (0.63) *
10–14 years	18.5 (5)	5.3 (1)	12.5 (5)	13.9 (10)	1.75 (0.63) *	0.03 (0.86) *
15–18 years	9.7 (3)	8.3 (1)	5.7 (2)	11.1 (4)	NA (0.90) ^#^	0.01 (0.93) *
Muscular involvement						
0–4 years	3.7 (5)	1 (1)	1.2 (3)	0.4 (3)	13.72 (0.003) *	11.56 (<0.001) *
5–9 years	7.1 (1)	0 (0)	3.6 (1)	3.8 (3)	0.73 (0.87) *	0.09 (0.76) *
10–14 years	3.7 (1)	0 (0)	5 (2)	5.6 (4)	1.16 (0.76) *	0.50 (0.48) *
15–18 years	22.6 (7)	8.3 (1)	0 (0)	5.6 (2)	11.22 (0.01) *	7.33 (0.007) *

Note: * These values should be interpreted with caution as they do not meet the reporting criteria for the chi-square test. ^#^ The *p*-value is derived from the Fisher exact test.
